# Socioeconomic, demographic and geographic determinants of food consumption in Mexico

**DOI:** 10.1371/journal.pone.0288235

**Published:** 2023-10-17

**Authors:** Louise Guibrunet, Ana G. Ortega-Avila, Esperanza Arnés, Francisco Mora Ardila

**Affiliations:** 1 Institute of Geography, National Autonomous University of Mexico, Mexico City, Mexico; 2 Research Centre on Environmental Geography, National Autonomous University of Mexico, Morelia, Mexico; 3 Research Institute on Ecosystems and Sustainability, National Autonomous University of Mexico, Morelia, Mexico; Hospital Infantil de México Federico Gomez: Hospital Infantil de Mexico Federico Gomez, MEXICO

## Abstract

What people eat affects public health and human wellbeing, agricultural production, and environmental sustainability. This paper explores the heterogeneity of food consumption patterns in an ecologically and culturally diverse country. Using a latent class approach (which creates clusters of individuals with homogeneous characteristics), we analyse a food questionnaire (from the National Health and Nutrition Survey) applied across Mexico. We identify four clusters of food consumption (staple, prudent, high meat and low fruit) and find that belonging to these clusters is determined by socioeconomic, demographic (age, sex) and geographic (region, urban/rural) characteristics. Maize and pulses tend to constitute a larger proportion of the diet of poor, rural populations living in the south, while urban populations eat more varied foods, including ingredients whose production systems tend to exert more pressure on natural resources (for instance, meat). Despite the importance given in the literature to the Mexican gastronomy and its diverse traditional regional diets, we find that only 6% of the population adopts a food consumption pattern resembling the traditional Mexican diet. Instead, most of the Mexican population has a food consumption pattern resembling a western diet, which is problematic in terms of public health and environmental sustainability.

## 1. Introduction

What people eat affects public health and human wellbeing; it is determinant for environmental planning and sustainability, and thus for the design of public policies related to food. Malnutrition is the world’s highest risk factor for disease [1]. Between 702 and 828 million people in the world suffered from hunger in 2021, yet at the same time more than 675.7 million suffered from obesity [2]. Diet is also determinant in the development of cardiovascular diseases, hypertension, diabetes and certain types of cancer [3, 4]. In parallel, the conventional agro-food system is a major driver of environmental degradation, causing land use change, greenhouse gas emissions and loss of biodiversity [5]. Inadequate food consumption, however, results from unequal food distribution and access, rather that insufficient production [6]. Achieving a sustainable food system thus implies profound and simultaneous changes to agricultural production systems, food distribution practices, and diets [4].

Diets are shaped by complex interactions of local biocultural diversity [7, 8], individual behaviour and cultural preferences [9], socioeconomic factors [10] as well as the built environment [11, 12] and international markets [10]. The globalisation of food markets has reduced the diversity of local diets and contributed to their homogenisation as the same products become available everywhere [13, 14]. However, diets remain marked to some extent by local characteristics, as illustrated by the persistence and importance of diverse local maize varieties in Mexico [15, 16].

Public policies must take into account how people eat across different social, economic, demographic and geographic contexts if a healthy and sustainable food system is to be achieved [17, 18]. Indeed, food policies can enhance public health; for instance, taxes on unhealthy foods are proven to be effective to tackle obesity [19]. However, ill-designed policies can have adverse effects; as shown in the case of Egypt, where food subsidies to address undernutrition have increased rates of obesity [20]. Failure to adapt national policies to (subnational) regional contexts may thus hinder the transition towards healthy and sustainable food systems.

In this paper, we focus on Mexico, a megadiverse country in terms of both ecosystems and cultures [21]. This case study can yield important insights to explore the case of other megadiverse countries (both in terms of biodiversity and sociocultural contexts). Despite the global tendency towards dietary homogenisation [22, 23], diets in Mexico differ across regions and social groups [24]. Dietary differences between the richest and poorest deciles of the Mexican population are particularly striking for meat consumption and overall caloric intake [25]. A regional study noted that meat consumption is higher among Mexican urban dwellers than rural dwellers [26]. Mexican rural diets are also heterogeneous, reflecting the diversity of species and varieties grown in the country’s regions [27]. Yet, national scale studies of food consumption in Mexico remain scarce.

The objective of the present study is to identify clusters of food consumption across Mexico and to associate them with socioeconomic, demographic and geographic determinants. Using a latent class approach on a food questionnaire applied across Mexico, we identify four clusters of food consumption (staple, prudent, high meat and low fruit). We discuss variations in food consumption across those clusters and conclude with implications for future research.

## 2. Methods

### 2.1 Data source

We explored food consumption in Mexico by using the 2018–2019 National Health and Nutrition Survey (ENSANUT). This survey is funded by the federal government and published periodically by two Mexican governmental bodies, the National Institute of Geography and Statistics (INEGI) and the National Institute of Public Health (INSP). The full database is publicly available online [28].

**Fig 1:**
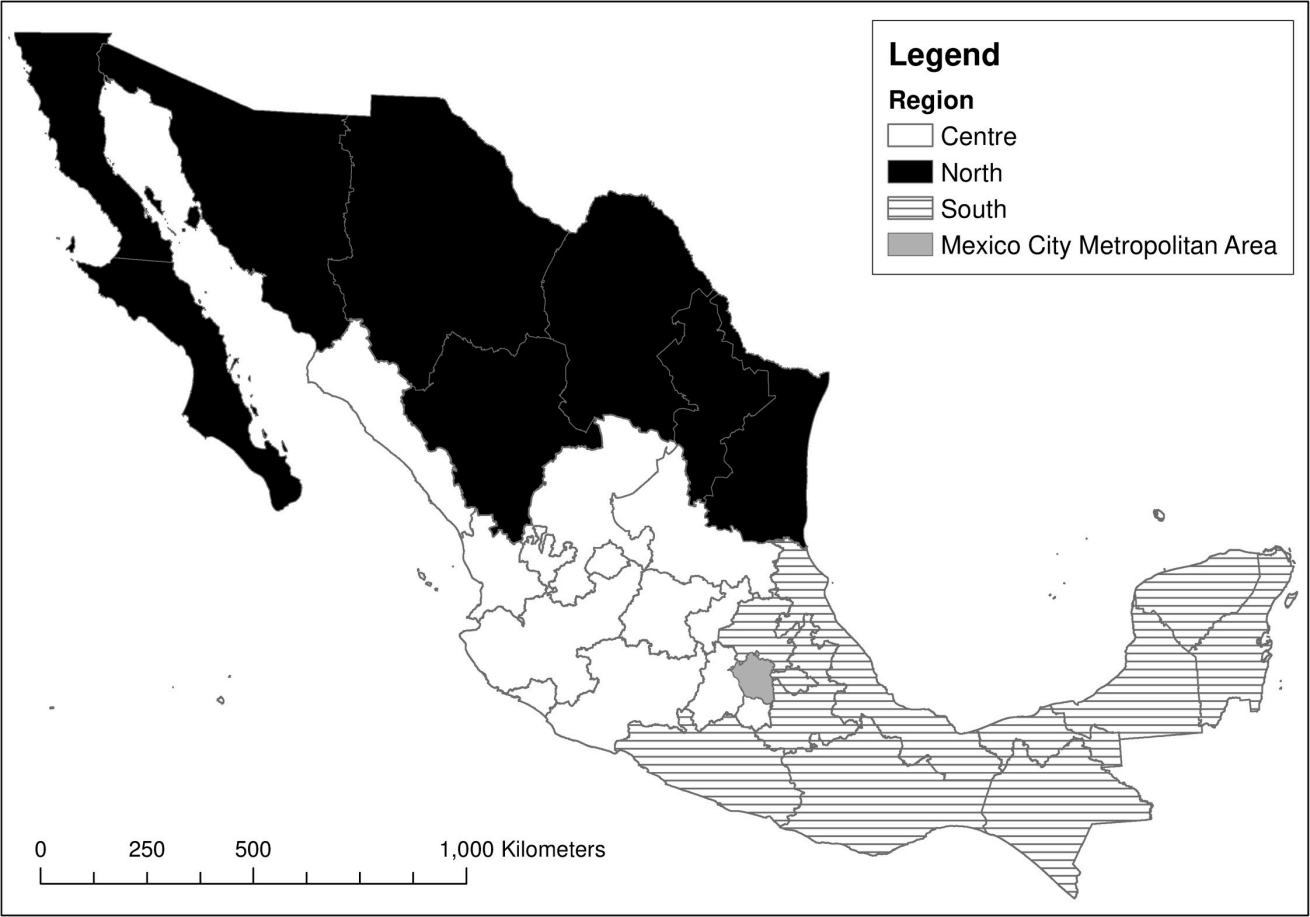
Division of Mexico into four regions proposed by ENSANUT. Map produced by authors using ENSANUT methodological document and governmental geomatic datasets [29, 34].

The nutrition survey was applied from the 30^th^ of July 2018 to the 15^th^ of February 2019 in respondents’ homes; it was administered to 32,079 individuals across Mexico. All variables were documented for all respondents; as such, there is no missing data for any respondent. The survey is cross-sectional, probabilistic, multi-stage and stratified, and it is representative of the Mexican population at the national level, regional level, and by type of settlement (urban/rural) [29, 30]. The regions proposed by ENSANUT and used in this paper (Fig 1) broadly reflect the climatic and cultural differences across the country which have shaped diverse diets [31–33].

The ENSANUT survey adhered to the Declaration of Helsinki and was granted ethics approval by the INSP ethics committee [30, 35]. We were not required by our institution to seek further ethics approval for our study, given that it uses the survey as secondary data.

### 2.2 Dietary assessment

ENSANUT documents what people have eaten in a week using a semi-quantitative Food Frequency Questionnaire (FFQ). The FFQ collects dietary information by asking the frequency of consumption (number of days and numbers of times per day individuals consumed specific food items) and quantity consumed, using standardised portions in grams or millilitres. The FFQ covers 140 foods, from fruits and vegetables to snacks and prepared meals (for instance, a *quesadilla* or a burger). Outliers were removed by the governmental agency before the publication of the data [29]. The FFQ was administered to 32,079 individuals across Mexico. However, for the purpose of this study, we excluded from the analysis children younger than 12 years of age. This is because the incorporation of children in our analysis would have affected the cluster identification of other age groups since children would have formed their own cluster. Also, we excluded pregnant and lactating women because food intake increases during pregnancy and lactation. This is consistent with other studies which apply the same exclusion criteria [24, 36]. Thus, the final sample consisted of 22,630 individuals.

We translated each portion of food consumed into its equivalents in dry weight of fourteen major food groups adapted from FAOSTAT (S1 Text). We used traditional Mexican recipes found online to translate food portions to their equivalent in terms of basic ingredients in dry weight. As an example, a portion of hamburger was translated to grams of meat, vegetables, dry flour, oil and cheese. Water (as an ingredient for the bread) was excluded (this method has been published previously [37]). Using dry weight has two advantages: firstly, it standardises food measurements and hence allows the summation of the intake of the same ingredient in different forms (for instance, maize consumed as corn on the cob, in soup or in *tortillas*). Secondly, the use of dry weight means that the data from the present study can be used to estimate the environmental impacts of food supply in future research (which cannot be done with calories as the measurement unit).

### 2.3 Socioeconomic, demographic and geographic variables

ENSANUT provides data on respondents’ sex (male or female), age, region of residence (north, centre, Mexico City Metropolitan Area–hereinafter “Mexico City” ‐ and south), and type of settlement (urban/rural). In addition, it also documents socioeconomic variables which can be used to estimate respondents’ socioeconomic status. We classified each respondent according to socioeconomic status by applying an existing method which recognises seven socioeconomic groups for Mexican households based on the number of bedrooms and bathrooms, number of cars/vans, internet connection, educational level of head of household and employment level [38].

### 2.4 Statistical analyses

Descriptive statistics were used to visualise average per capita food consumption across socioeconomic, demographic and geographic characteristics. Weighted averages were calculated to estimate average per-capita food consumption in a representative manner. To do so, we multiplied individual consumption by respondents’ sampling weights; we then divided those numbers by the sum of the sampling weights. The sampling weights were included in the original database published by the government, and were provided to make the data representative of the Mexican population.

To identify clusters of food consumption, we used Latent Profile Analysis (LPA), a statistical procedure that assigns observations to clusters based on their responses to a series of continuous variables (indicators) and possibly conditioned on covariates that help to predict their latent profile membership [39, 40]. It assumes the existence of unobserved latent profiles that generate patterns on the indicators, and uses a probabilistic model to derive the clusters [41]. LPA was chosen instead of standard cluster analysis techniques (e.g. K-means or hierarchical clustering) because it allows selection of the number of profiles based on goodness of fit or information criteria, thereby reducing the subjectivity in the definition of the number of profiles that would be associated with classical clustering techniques.

We used the relative individual consumption of fourteen food groups as continuous indicators. This was calculated by dividing the individual’s food group consumption by the individual’s total food consumption (both in grams/day). This allows us to explore the extent to which certain food groups are incorporated into an individual’s diet and to avoid clustering based on the total amounts of food consumed, which is heavily determined by age, sex and body mass. The five socioeconomic, demographic and geographic variables (socioeconomic status, age, sex, region, and type of settlement) were used as covariates (S1 Fig).

We ran five models with an increasing number of classes (S2 Text) and used an entropy criterion, which measures the quality of classification (i.e., the probability that participants belong to one of the classes) [42], to determine the optimal number of classes. The second step in LPA fitting was the inclusion of covariates, for which we followed the 3-step approach [43]. The procedure consists in enumerating the classes, followed by creating the most likely class variable using the latent class posterior distribution (obtained during the first step), and then regressing the most likely class on predictor variables, in this case using a multinomial logistic regression. This resulted in the relative risk ratio of being allocated to a certain cluster in relation to the covariates. The analysis was conducted in R version 4.1.3 and Mplus version 8.8.

## 3. Results

### 3.1 Characteristics of respondents and food consumption patterns

The sample of the individuals included in the current study (Table 1) was composed of a higher proportion of women than men, a higher proportion of teenagers and young adults (18–29) than other age groups, and a majority of urban dwellers, thus representing the general demographic patterns of Mexico [44].

**Table 1:** Sociodemographic characteristics of the individuals included in the study.

	Number	Percentage
Total population	22,630	100%
*Sex*		
Male	10,370	46%
Female	12,260	54%
*Type of settlement*		
Urban	14,941	66%
Rural	7,689	34%
*Region *		
North	5,072	22%
Centre	8,446	37%
Mexico City	700	3%
South	8,412	37%
*Socioeconomic status*		
E (lowest)	1,038	5%
D	7,274	32%
D+	4,363	19%
C-	4,002	18%
C	3,393	15%
C+	2,081	9%
A/B (highest)	479	2%
*Age*		
12–17	4,449	20%
18–29	4,330	19%
30–39	3,488	15%
40–49	3,525	16%
50–59	2,722	12%
60–69	2,122	9%
70–79	1,309	6%
80+	685	3%

**Fig 2:**
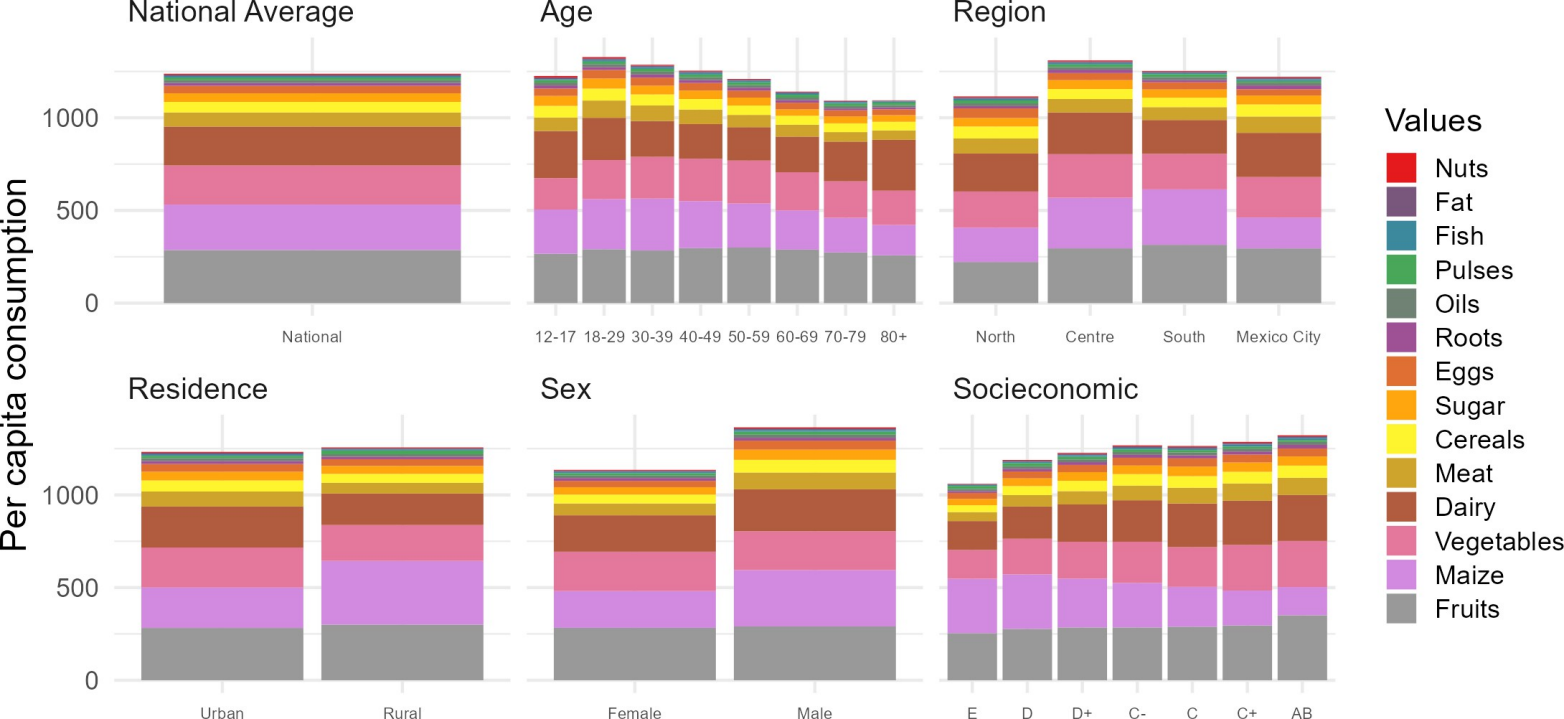
Food consumption in Mexico, 2018–2019, across socioeconomic, demographic and geographic characteristics. Average per capita consumption. Grams per day. The data plotted in this figure can be found in S1 Table. Figure produced by authors.

Per capita food consumption differs across socioeconomic, demographic and geographic characteristics (Fig 2). The total quantity of food consumed varied across socioeconomic groups, with individuals of the highest socioeconomic status consuming 20% (261 grams) more food daily than individuals of the lowest socioeconomic status. Individuals of lower economic status tended to eat more pulses and maize (15% to 20% more than the national average for the two lowest groups). The consumption of all other foods (fruit, vegetables, dairy, meat, fat, cereals, sugar, roots, oil, fish and nuts) increased with socioeconomic status. In particular, the consumption of vegetables and cereals other than maize varied with socioeconomic status (the individuals of the highest status eating on average 37% more vegetables than those of the lowest status).

The quantity and type of food consumed both differed according to age and sex. For instance, men consumed 17% (229 grams) more food per day than women. Women incorporated more fruit and vegetables as a proportion of their total food consumption, and relatively less maize. Meat formed an important part of the diet for people aged 18–39 whereas pulses formed a more important part of the diet for people aged 50–69 (S1 Table).

Rural residents ate 37% more maize and 36% more pulses than urban residents, and consumed less meat (-39%), fish (-36%) and dairy (-31%). Urban residents ate 24% more (non-maize) cereals than rural residents. Food consumption also differed across the country: while residents of the north of the country ate 7% more meat and fish, 27% more eggs and 14% more cereals (excluding maize) than the national average, residents of the south and centre of the country ate more maize (11% and 22% respectively), fruits (3% and 10%) and pulses (1% and 10%) than the national average. Mexico City residents consumed 31% more roots, 16% more meat, 14% more cereals and 13% more dairy than the national average.

### 3.2 Clusters of food consumption

**Fig 3:**
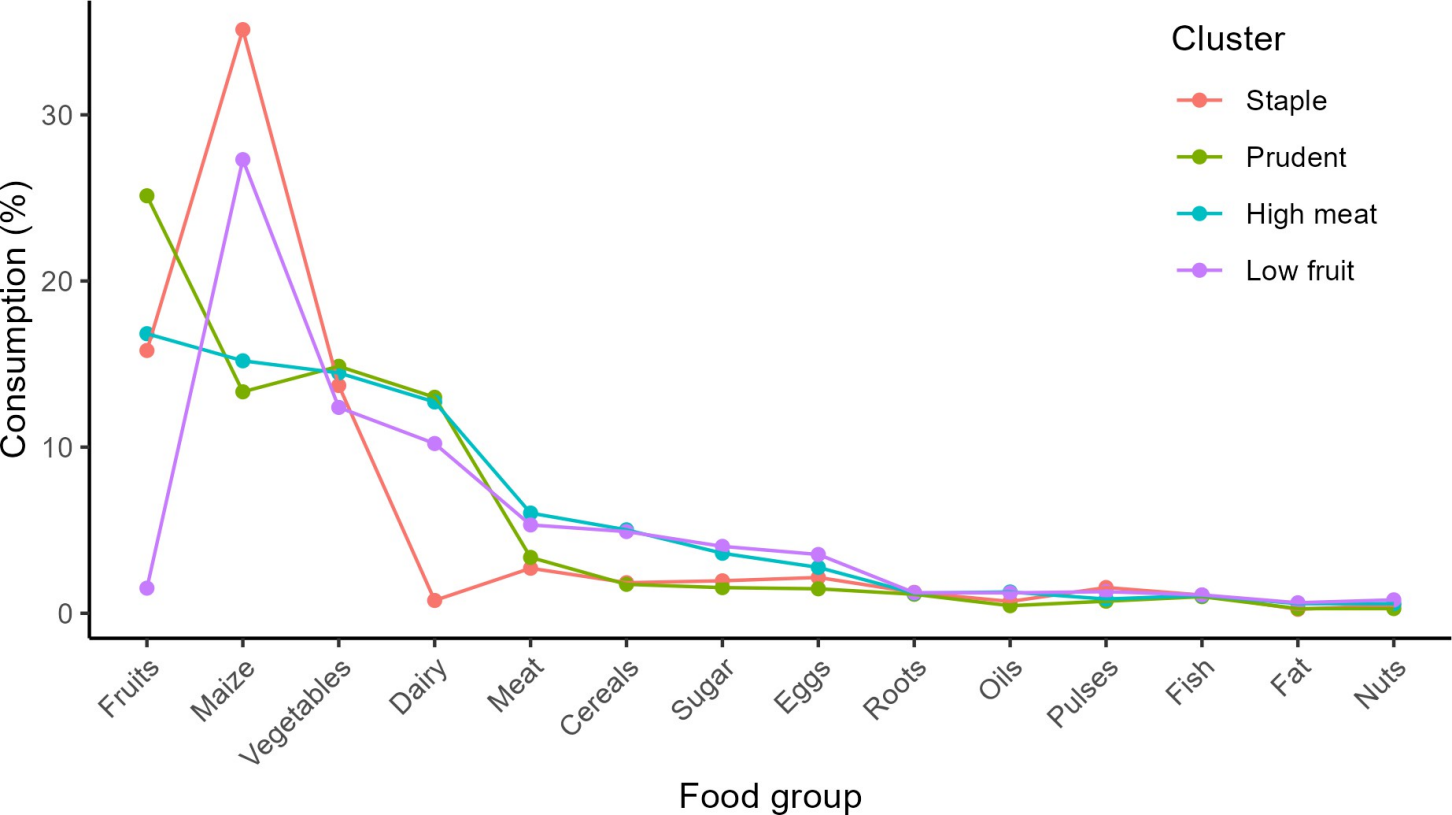
Relative consumption of each food group for the four clusters. Relative consumption is calculated as the share of each food group in total individual consumption. The data plotted in this figure is presented in S2 Table. Figure produced by authors.

The LPA suggests that food consumption patterns can best be grouped into four distinct clusters (S2 Text and Fig 3). The “staple” cluster is characterised by a relatively higher proportion of maize and pulses in total food consumption; it represents 6% of the population. The “prudent” cluster consumes relatively more fruits and vegetables and dairy and incorporates the different food groups in a balanced manner; it represents 26% of the population. The “high meat” cluster consumes relatively more meat and cereals and represents 60% of the population. Finally, the “low fruit” cluster consumes more eggs, oil, fats and sugar and less fruit, meat, and vegetables; it represents 8% of the population.

### 3.3 Determinants of food consumption patterns

**Fig 4:**
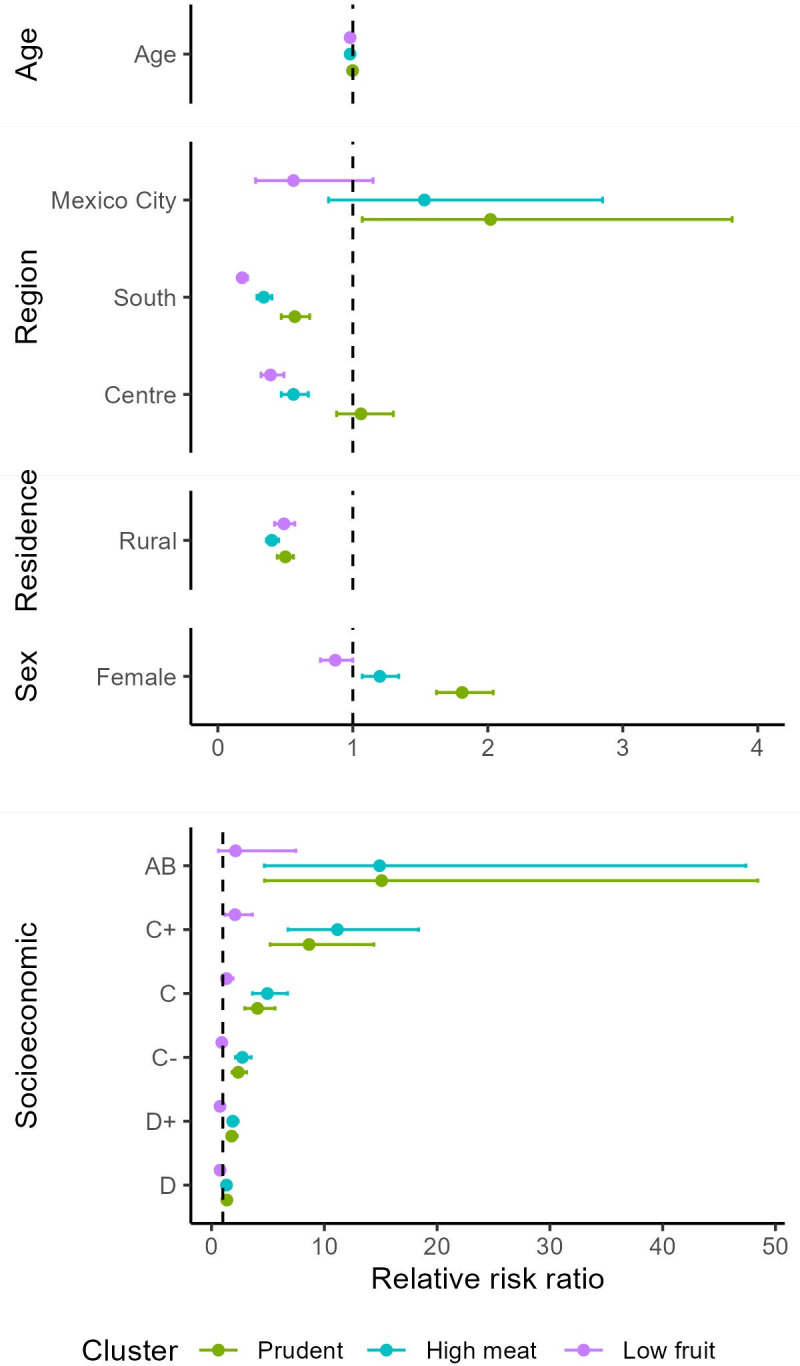
Multinomial logistic regression using the staple cluster as reference. Relative risk ratios (points) and their 95% confidence intervals (bars) are shown for each of the categories within the socioeconomic, demographic and geographic location covariate used in the study. For each covariate, a reference level was set to calculate risk ratios: north (region), urban (type of settlement), male (sex), and group E (the lowest) (socioeconomic status). For age, risk ratios were calculated as the ratio of slopes. All the values are relative (risk values) as they are also relative to the values of the staple cluster. Confidence intervals not crossing the dashed line indicate relative risk ratios that differ significantly from that of the staple cluster. Data is presented in S3 Table. Figure produced by authors.

Belonging to a given cluster was significantly associated with socioeconomic, demographic and geographic characteristics (Fig 4). For females relative to males, the relative risk of being in the prudent cluster relative to the staple cluster increased by a factor of 1.8. Those living in the south and in Mexico City relative to those in the north were more likely to be in the prudent cluster relative to the staple cluster. Those residing in rural areas relative to urban dwellers were less likely to be in the prudent cluster relative to the staple cluster. In terms of socioeconomic status, groups D to A relative to level E (lowest) were more likely to be in the prudent cluster relative to the staple cluster.

With an increase in age by one unit, the relative risk of being in the high meat cluster relative to the staple cluster decreased by a factor of 0.98. Females relative to males, were more likely to be in the high meat cluster relative to the staple cluster. Those living in the centre and south regions relative to the those living in the north, were less likely to be in the high meat cluster relative to the staple cluster. For those residing in rural areas relative to urban areas, the relative risk for being in the high meat cluster relative to the staple cluster decreased by a factor of 0.40. All socioeconomic groups relative to level E were more likely to be in the high meat cluster relative to the staple cluster.

With an increase in age by one unit, the relative risk of being in the low fruit cluster relative to the staple cluster decreased by a factor of 0.98. Those living in the centre and south regions relative to the those living in the north, were less likely to be in the low fruit cluster relative to the staple cluster. For those residing in rural areas relative to urban areas, the relative risk for being in the low fruit cluster relative to the staple cluster decreased by a factor of 0.49. Those in socioeconomic group D- relative to those in group E were less likely to be in the low fruit cluster relative to the staple cluster, however those in group B were more likely to be in the low fruit cluster relative to the staple cluster.

From this analysis, the following insights emerged: the staple cluster was mainly composed of people living in rural areas and in the south of the country, relatively older and poorer than the rest of the population. In relation to the staple cluster, the prudent and high meat clusters were more likely to be composed of women living in Mexico City and belonging to any socioeconomic group other than the lowest. People belonging to the low fruit cluster (relative to the staple cluster), were likely younger, of higher socioeconomic status, and living in the north of the country.

## 4. Discussion

Our analysis shows food consumption patterns in Mexico to be diverse and significantly associated with socioeconomic, demographic and geographic factors. The LPA identifies four clusters that differ in terms of the consumption of meat, dairy products, maize, pulses, fruits and vegetables. We discuss these insights in more detail below.

### 4.1 Most of the Mexican population has a food consumption pattern resembling a western diet

The food consumption of the staple cluster is the one that most resembles the traditional Mexican diet, which is characterised by a high consumption of maize, vegetables and pulses and a low consumption of animal proteins [45]. However, this cluster only represents 6% of the population (mostly elderly, poor and rural), suggesting that this diet is no longer prevalent across the Mexican population. This is problematic, given that the traditional diet has health benefits [46] and contributes to the preservation of local agroecosystems [47].

On the contrary, two clusters (high meat and low fruit) resemble the western dietary pattern, which is characterised by high consumption of sugars, salt, and saturated fat, generally in the form of processed food, and by protein intake primarily from red meat [48]. The western dietary pattern has been associated with negative health outcomes such as type 2 diabetes, obesity, hypertension and cancer [49, 50]. Additionally, this consumption pattern is more resource-intensive in the production stage, and thus more problematic from the perspective of environmental sustainability [51, 52]. The high meat and low fruit clusters represent more than two thirds (68%) of the population, particularly the youth and urban dwellers. This may indicate that as the country urbanises, more and more people may move towards a diet that is harmful for both health (due to high consumption of added sugar and saturated fat) and the environment (with a protein intake mostly from animal products). This pattern has been observed in studies of other countries across the world, where urbanisation is correlated with increases in consumption of meat and non-staple products (such as sugar or oil) as a share of total food intake [52], and particularly for urban dwellers of middle to upper socioeconomic status [53].

### 4.2 The traditional Mexican diet is adopted mainly by elderly people

We find that young and middle-aged adults (18 to 39 years old) eat relatively more meat, animal fats and sugars than do elderly adults. Pulse consumption of individuals under 29 years old is the lowest. This echoes Castellanos-Gutiérrez et al.’s (2021) study which finds that pulse consumption in Mexico is lower than is recommended for a healthy and sustainable diet [24]. Within our sample, the older cohorts had a higher consumption of pulses and a lower consumption of fats, oils and sugars. This suggests that the young population is increasingly adopting a western diet and abandoning staples that make up the traditional Mexican diet.

### 4.3 Food consumption varies across socioeconomic status

Our results show that socioeconomic status is a determinant of food consumption, with individuals of higher socioeconomic status consuming more food in general, and particularly more meat, whereas individuals belonging to the lower socioeconomic groups consume more maize and pulses (cheaper staple foods). This is consistent with data from Mexico City [54] that suggests that the traditional dietary pattern (consisting primarily of maize and beans) is associated with lower socioeconomic status. These results also coincide with studies conducted in other countries where higher socioeconomic status is associated with higher consumption of calories and animal-based foods [55, 56]. A study of 2006–2016 data has suggested that Mexican adults with lower socioeconomic status have a better quality diet than those with higher socioeconomic status [36]. Although there is some indication in our data that groups of lower socioeconomic status have relatively healthier and more traditional diets, we also found that the staple food cluster, characterised by a concentration of individuals of lower socioeconomic status, incorporates less animal proteins (than do other clusters), whether the source be dairy, eggs, fish or meat. Although this consumption pattern implies low greenhouse gas emissions (mostly associated with red meat and dairy production) [57], it may be detrimental to health. Previous studies have suggested that although high consumption of animal-based food, especially red and processed meats, are not recommended due to causing cardiovascular disease and dyslipidaemia, intake of animal protein from sources such as fish, poultry and dairy in combination with plant based-proteins can help reach dietary protein requirement [58]. Protein deficiency can lead to cardiovascular dysfunction, a high risk of infectious disease, deficiency of other nutrients (including vitamin A and iron) and impaired metabolic profiles [59].

### 4.4 Food consumption reflects regional biocultural diversity

In Mexico and elsewhere, the region and type of settlement are determinant in the diet [26, 60]. Our analysis demonstrates that Mexico’s biocultural diversity is reflected in diverse food consumption patterns in different regions of the country. In particular, meat consumption is higher in the north of the country, a semi-arid ecoregion characterised by traditional extensive livestock farming [61]. The central region, where maize represents an important share of the diet, is where most of the country’s maize is produced [62, 63]. Fruit consumption is slightly higher in the southern region, where production is higher and where climate allows for a greater variety of fruit species. Although the production of fruit and vegetables in Mexico exceeds consumption [64], this is only reflected in a higher consumption of fruit (6%) by the rural population, but not in a higher consumption of vegetables (which is 11% lower for the rural population). This can be explained by the high volume of fruits and vegetables produced for sale and export (cash crops) instead of for national consumption, especially in the centre of the country [65]. Residents of the central and southern regions of the country, where the *milpa* (The milpa is a traditional agricultural system from central Mexico which intercrops maize, beans, squash and chili, among other species.) is most common, incorporate more pulses into their diets than those of Mexico City and the north. The heterogeneity of food consumption patterns across Mexican regions suggests a strong adherence to local traditional diets, despite the increasing homogeneity of food offer within and across countries [13, 14, 22]. Given that local traditional diets tend to be more resource-efficient, nutritious and culturally appropriate [66–68] and can have lower greenhouse gas emissions associated with transport [69], they can contribute to a healthy and sustainable food system [17, 18]. For instance, the consumption of *milpa* products can improve the nutrition of the population, conserve the gastronomic heritage and sustain agrobiodiversity [70, 71].

### 4.5 Strengths and limitations of our study

The first limitation of our study is that our analysis is based on dietary information which was self-reported, which increases the chance of recall bias [72], measurement errors and under-estimations [37, 73]. An additional source of uncertainty is the conversion of food portions to dry weight of different food groups: this method fails to incorporate the likely variations in the recipes and food preparation techniques which vary across individuals and which imply differences in the ingredients used and their quantities. Another limitation is that of the “naming fallacy”, which is when researchers assign names to clusters which do not accurately reflect the content of the cluster [40]. We are aware that, owing to the subjectivity in assigning names to the clusters, we could commit the naming fallacy. To mitigate this risk, we provide detailed information about the clusters so that readers can easily identify the composition of each cluster and avoid oversimplification of the study results. Our study focuses on the case of Mexico; its results cannot be generalised beyond the Mexican context. However, our method can be replicated and applied to other cases.

Among the strengths of this paper, our study is the first, to our knowledge, to analyse food consumption patterns in Mexico by using a latent class approach. Since this statistical method reduces bias in the determination of the clusters, it provides robust results. Moreover, we analysed food consumption using dry weight instead of caloric intake. This method allows to conclusions to be reached not only with nutrition in mind, but also in terms of total food supply needed in different regions of the country and the associated environmental impacts of food consumption. This method is therefore pertinent as an input for policy making, and the data presented in this paper can be used in further research on the topic.

## 5. Conclusion

This study uses a latent class approach to analyse data from a food questionnaire applied throughout Mexico. It identifies four distinct clusters of food consumption; belonging to those clusters is significantly associated with socioeconomic, demographic and geographic factors. Despite the importance given in the literature to the Mexican gastronomy and its diversity of traditional regional diets [46, 71, 74], we find that a small share of the population adopts a consumption pattern based on staple foods from the traditional Mexican diet (maize and beans). Instead, most of the Mexican population has a food consumption pattern resembling a western diet, which is problematic in terms of public health and environmental sustainability [49, 51]. An understanding of the variation of food consumption across socioeconomic, demographic and geographic contexts is therefore essential for the design of effective and locally pertinent public policy to achieve universal consumption of healthy, nutritious and environmentally friendly diets. Specific policy alternatives, tackling socioeconomic, demographic and geographic factors associated with food consumption should be tested through further research.

## Supporting information

S1 Text List of food groups used in the analysis. (PDF)

S2 Text Model fit statistics. (PDF)

S1 Fig Path analysis of the LPA. (TIF)

S1 Table Data plotted in Fig 2. Average per capita consumption (g/day) across socioeconomic, demographic and geographic characteristics.
(PDF)

S2 Table Data plotted in Fig 3. Average relative consumption of each food group (as share of total individual consumption) for the four clusters identified through the LPA.
(PDF)

S3 Table Data plotted in Fig 4. Multinomial logistic regression using the staple cluster as reference.
(PDF)
