# Correction: Socioeconomic, demographic and geographic determinants of food consumption in Mexico

**DOI:** 10.1371/journal.pone.0306437

**Published:** 2024-06-27

**Authors:** Louise Guibrunet, Ana G. Ortega-Avila, Esperanza Arnés, Francisco Mora Ardila

There is an error in affiliation 2 for author Esperanza Arnés. The correct affiliation 2 is: CONAHCYT—Research Centre on Environmental Geography, National Autonomous University of Mexico, Morelia, Mexico.

## References

[1] Guibrunet L, Ortega-Avila AG, Arnés E, Mora Ardila F (2023) Socioeconomic, demographic and geographic determinants of food consumption in Mexico. PLoS ONE 18(10): e0288235. 10.1371/journal.pone.0288235 37847715 PMC10581491

