# Supplementary material for: Socioeconomic, demographic and geographic determinants of food consumption in Mexico
Source: PLoS One. 2023 Oct 17;18(10):e0288235. doi: 10.1371/journal.pone.0288235 (PMC10581491; doi:10.1371/journal.pone.0288235)
Supplement: S1 Text — (PDF) [file pone.0288235.s001.pdf]

S1 Text. List of food groups used in the analysis

The list below provides the full list of food items considered in each food group. We used the FAOSTAT categorisation to enhance comparability with other studies. We excluded some groups for which ENSANUT provides no data (spices, oil crops, aquatic products) as well as two other groups, alcoholic beverages and stimulants (coffee and tea), that do not refer to food consumption. We merged the “meat” and “offal” categories. Finally, we separated maize from other cereals, as has been done in other studies on Mexico [1] because of its importance as a staple food in the country: for instance, maize represents 23% of the caloric intake of Mexican rural women [2].

Food types included in each food group:

Cereals: Barley; Millet; Oats; Rice; Rye; Sorghum; Wheat; Other cereals (excluding beer)

Dairy: Milk (excluding butter), yoghurt, cheese

Eggs: Eggs

Fats: Butter, Ghee; Cream; Raw animal fats; Fish oils

Fish: Fish; Cephalopods and other molluscs; Crustaceans; Molluscs; Other seafood

Fruits: Apples; Bananas; Citrus; Dates; Grapefruit; Grapes (excluding wine); Lemons, Limes; Oranges, Mandarines; Pineapples; Plantains; Other fruits

Maize: Maize

Meat: Bovine Meat; Mutton & Goat Meat; Pigmeat; Poultry Meat; Edible offal; Other meat
Nuts: Nuts and related products
Oils: Vegetable oils including coconut, cottonseed, groundnut, maize, olive, palm, rice, soybean, sunflower and others; Oilcrops
Pulses: Beans; Peas; Other pulses (including lentils).
Roots: Starchy roots and tubers: Cassava; Potatoes; Sweet potatoes; Yams; Other roots
Sugar: Honey; Sugar; Sweeteners
Vegetables: Onions; Tomatoes; Other vegetables

References cited in S1 Text:

1. Castellanos-Gutiérrez A, Sánchez-Pimienta TG, Batis C, Willett W, Rivera JA. Toward a healthy and sustainable diet in Mexico: Where are we and how can we move forward? Am J Clin Nutr. 2021;113: 1177–1184. doi:10.1093/ajcn/nqaa411

2. Benítez-Arciniega AD, Vizcarra-Bordi I, Ochoa-Rivera T, del Carmen Guzmán-Márquez M, Morales-González L, Hernández-Ramírez J. Consumption of maize food group and its contribution on the diet of rural women in México. Proc Nutr Soc. 2020;79: 2020. doi:10.1017/s0029665120003699
