# Supplementary material for: Socioeconomic, demographic and geographic determinants of food consumption in Mexico
Source: PLoS One. 2023 Oct 17;18(10):e0288235. doi: 10.1371/journal.pone.0288235 (PMC10581491; doi:10.1371/journal.pone.0288235)
Supplement: S2 Text — (PDF) [file pone.0288235.s002.pdf]

S3 Text. Model fit statistics

To select the number of profiles as part of the LPA, we used model fit statistics such as Akaike Information Criterion (AIC), Bayesian Information Criterion (BIC), and sample size adjusted BIC (SSABIC) (see Table S3). Based on model fit statistics, entropy suggests that the model with 4 classes has the largest coefficient with 0.81. Nonetheless, AIC, BIC and SSABIC indicate that the model with 6 classes has the lowest coefficient. Considering there is a discrepancy with the information criteria statistics, we decided to explore further the distribution of individuals, specifically the proportions of individuals within each class, as well as the distribution of food group consumption. This, to make sure that the number of classes did not create classes so small that they lacked no meaningful content. To select the most appropriate number of classes we explored the graphical representation of the probabilities of belonging to each class of the 4 class model and the 6 class. We noticed that even when model fit statistic suggested the 6 classes model, the model divided some classes in two. Therefore, we decided to select the 4 class model mainly based on entropy.

	AIC	BIC	SSABIC	Entropy
2 classes	753001.8	753347	753210.3	0.71
3 classes	746625.9	747091.5	746907.2	0.79
4 classes	742011.7	742597.7	742365.7	0.81
5 classes	738857.9	739564.3	739284.6	0.76
6 classes	735301.9	736128.7	735801.3	0.79

Table S3. Model fit statistics
