# Supplementary figures and images for: Socioeconomic, demographic and geographic determinants of food consumption in Mexico
Source: PLoS One. 2023 Oct 17;18(10):e0288235. doi: 10.1371/journal.pone.0288235 (PMC10581491; doi:10.1371/journal.pone.0288235)

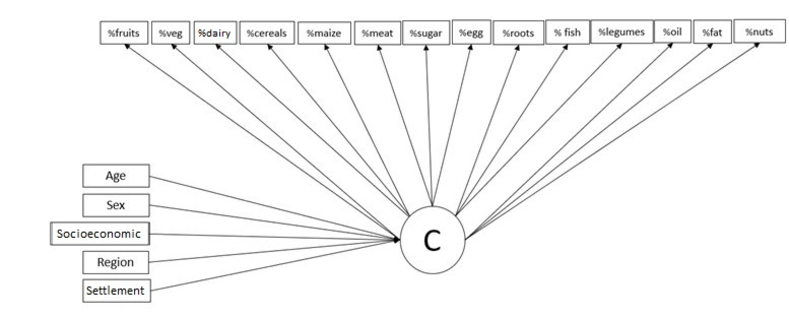

Supplement: S1 Fig — (TIF) [file pone.0288235.s003.tif]
