# Supplementary material for: Socioeconomic, demographic and geographic determinants of food consumption in Mexico
Source: PLoS One. 2023 Oct 17;18(10):e0288235. doi: 10.1371/journal.pone.0288235 (PMC10581491; doi:10.1371/journal.pone.0288235)
Supplement: S1 Table — Average per capita consumption (g/day) across socioeconomic, demographic and geographic characteristics. (PDF) [file pone.0288235.s004.pdf]

**S4 Table. Data plotted in Fig 2.** Average per capita consumption (g/day) across socioeconomic, demographic and geographic characteristics

| **Food group** | | **Fruits** | **Maize** | **Vegetables** | **Dairy** | **Meat** | **Cereals** | **Sugar** | **Eggs** | **Roots** | **Oils** | **Pulses** | **Fat** | **Fish** | **Nuts** | **Total** |
|---|---|---|---|---|---|---|---|---|---|---|---|---|---|---|---|---|
| National Average | | 286 | 245 | 211 | 210 | 76 | 57 | 46 | 41 | 15 | 15 | 14 | 8 | 8 | 4 | 1,238 |
| By region | North | 222 | 185 | 195 | 205 | 81 | 65 | 46 | 52 | 12 | 15 | 15 | 8 | 9 | 5 | 1,115 |
| | Centre | 295 | 273 | 235 | 225 | 72 | 54 | 48 | 38 | 18 | 14 | 15 | 8 | 8 | 5 | 1,310 |
| | Mexico City | 295 | 167 | 218 | 238 | 88 | 65 | 48 | 34 | 20 | 15 | 9 | 8 | 9 | 5 | 1,221 |
| | South | 315 | 299 | 192 | 182 | 69 | 51 | 44 | 39 | 12 | 15 | 16 | 7 | 7 | 3 | 1,253 |
| By type of settlement | Urban | 282 | 218 | 216 | 221 | 81 | 60 | 47 | 42 | 16 | 15 | 13 | 8 | 9 | 5 | 1,232 |
| | Rural | 300 | 344 | 194 | 169 | 58 | 48 | 43 | 36 | 13 | 14 | 20 | 7 | 6 | 4 | 1,256 |
| By socioeconomic group | E | 254 | 293 | 156 | 156 | 48 | 37 | 34 | 32 | 10 | 11 | 17 | 5 | 5 | 2 | 1,061 |
| | D | 277 | 294 | 193 | 174 | 62 | 48 | 42 | 37 | 14 | 14 | 17 | 7 | 7 | 3 | 1,189 |
| | D+ | 285 | 264 | 199 | 201 | 72 | 56 | 47 | 39 | 15 | 15 | 15 | 8 | 8 | 4 | 1,227 |
| | C- | 285 | 240 | 222 | 224 | 79 | 62 | 46 | 42 | 16 | 15 | 14 | 9 | 9 | 5 | 1,267 |
| | C | 289 | 215 | 214 | 235 | 85 | 62 | 52 | 44 | 17 | 16 | 13 | 9 | 8 | 5 | 1,264 |
| | C+ | 296 | 188 | 246 | 239 | 93 | 63 | 50 | 43 | 16 | 15 | 11 | 9 | 11 | 6 | 1,286 |
| | AB | 351 | 153 | 249 | 248 | 93 | 66 | 50 | 43 | 19 | 16 | 10 | 9 | 11 | 5 | 1,322 |
| By age | 12-17 | 266 | 239 | 170 | 254 | 73 | 63 | 54 | 40 | 16 | 16 | 13 | 9 | 6 | 8 | 1,225 |
| | 18-29 | 291 | 270 | 211 | 228 | 93 | 65 | 54 | 47 | 16 | 16 | 13 | 9 | 9 | 6 | 1,327 |
| | 30-39 | 284 | 280 | 225 | 193 | 84 | 59 | 48 | 43 | 17 | 15 | 15 | 9 | 9 | 4 | 1,286 |
| | 40-49 | 297 | 253 | 229 | 187 | 79 | 57 | 46 | 41 | 16 | 15 | 15 | 8 | 9 | 3 | 1,254 |
| | 50-59 | 300 | 238 | 232 | 180 | 66 | 51 | 43 | 37 | 16 | 14 | 15 | 7 | 8 | 3 | 1,210 |
| | 60-69 | 289 | 211 | 206 | 192 | 64 | 49 | 34 | 36 | 15 | 13 | 15 | 7 | 8 | 2 | 1,141 |
| | 70-79 | 274 | 187 | 197 | 214 | 51 | 47 | 38 | 33 | 10 | 13 | 14 | 5 | 7 | 2 | 1,092 |
| | 80+ | 258 | 164 | 185 | 274 | 51 | 46 | 36 | 31 | 10 | 13 | 13 | 5 | 6 | 1 | 1,094 |
| By sex | Male | 290 | 304 | 209 | 227 | 91 | 68 | 55 | 49 | 15 | 17 | 16 | 9 | 9 | 5 | 1,365 |
| | Female | 283 | 198 | 212 | 196 | 64 | 48 | 40 | 34 | 15 | 13 | 13 | 7 | 7 | 4 | 1,135 |
