# Supplementary material for: Socioeconomic, demographic and geographic determinants of food consumption in Mexico
Source: PLoS One. 2023 Oct 17;18(10):e0288235. doi: 10.1371/journal.pone.0288235 (PMC10581491; doi:10.1371/journal.pone.0288235)
Supplement: S2 Table — Average relative consumption of each food group (as share of total individual consumption) for the four clusters identified through the LPA. (PDF) [file pone.0288235.s005.pdf]

**S5 Table. Data plotted in Fig 3.** Average relative consumption of each food group (as share of total individual consumption) for the four clusters identified through the LPA

| Food group | Staple | Prudent | High meat | Low fruit |
|---|---|---|---|---|
| Cereals | 2% | 2% | 5% | 5% |
| Eggs | 2% | 1% | 3% | 4% |
| Dairy | 1% | 13% | 13% | 10% |
| Fat | 0% | 0% | 1% | 1% |
| Fish | 1% | 1% | 1% | 1% |
| Fruits | 16% | 25% | 17% | 2% |
| Maize | 35% | 13% | 15% | 27% |
| Meat | 3% | 3% | 6% | 5% |
| Nuts | 0% | 0% | 1% | 1% |
| Oils | 1% | 0% | 1% | 1% |
| Pulses | 2% | 1% | 1% | 1% |
| Roots | 1% | 1% | 1% | 1% |
| Sugar | 2% | 2% | 4% | 4% |
| Vegetables | 14% | 15% | 14% | 12% |
