# Supplementary material for: Socioeconomic, demographic and geographic determinants of food consumption in Mexico
Source: PLoS One. 2023 Oct 17;18(10):e0288235. doi: 10.1371/journal.pone.0288235 (PMC10581491; doi:10.1371/journal.pone.0288235)
Supplement: S3 Table — Multinomial logistic regression using the staple cluster as reference. (PDF) [file pone.0288235.s006.pdf]

S6 Table. Data plotted in Fig 4. Multinomial logistic regression using the staple cluster as reference

	**Prudent cluster (26%)**					**High meat cluster (60%)**					**Low fruit cluster (8%)**				
	**RRR[e]**	**SE[f]**	**p-value[g]**	**95% CI[h]**		**RRR**	**SE**	**p-value**	**95% CI**		**RRR**	**SE**	**p-value**	**95% CI**	
**Age**	.998	0.00	0.23	[0.995	1.00]	0.98	0.00	**<0.001**	0.98	0.99	0.98	0.00	**<0.001**	0.98	0.99
**Sex[a] (female)**	1.81	0.11	**<0.001**	1.62	2.04	1.20	0.07	**0.001**	1.07	1.34	0.87	0.06	0.05	0.76	1.00
**Region[b]**															
Centre	1.06	0.11	0.51	0.88	1.30	0.56	0.05	**<0.001**	0.47	0.67	0.39	0.04	**<0.001**	0.32	0.49
Mexico City	2.02	0.66	**0.03**	1.07	3.81	1.53	0.49	0.18	0.82	2.85	0.56	0.20	0.11	0.28	1.15
South	0.57	0.05	**<0.001**	0.47	0.68	0.34	0.03	**<0.001**	0.29	0.40	0.18	0.02	**<0.001**	0.15	0.22
**Type of settlement[c] (rural)**	0.50	0.03	**<0.001**	0.44	0.56	0.40	0.02	**<0.001**	0.36	0.45	0.49	0.04	**<0.001**	0.42	0.57
**Socioeconomic level[d]**															
D	1.36	0.15	**0.005**	1.10	1.69	1.33	0.14	**0.007**	1.08	1.63	0.76	0.10	**0.03**	0.58	0.98
D+	1.78	0.22	**<0.001**	1.39	2.27	1.88	0.22	**<0.001**	1.49	2.36	0.75	0.11	0.05	0.56	1.00
C-	2.37	0.32	**<0.001**	1.82	3.10	2.73	0.35	**<0.001**	2.12	3.51	0.91	0.15	0.57	0.67	1.25
C	4.07	0.67	**<0.001**	2.95	5.63	4.95	0.78	**<0.001**	3.63	6.74	1.32	0.25	0.14	0.91	1.90
C+	8.65	2.25	**<0.001**	5.20	14.39	11.16	2.83	**<0.001**	6.78	18.35	2.08	0.59	**0.01**	1.19	3.62
A/B(richest)	15.08	8.98	**<0.001**	4.70	48.43	14.90	8.79	**<0.001**	4.68	47.37	2.13	1.36	0.24	0.61	7.47

[a]Reference is male; [b] reference is north; [c] reference is urban; [d] reference is E (lowest), [e]Relative Risk Ratios; [f] Standard Error; [g]significance level <0.05; [h] Confidence Intervals
